# Supplementary material for: Fluorescent Carbon Dots from Food Industry By-Products for Cell Imaging
Source: J Funct Biomater. 2023 Feb 7;14(2):90. doi: 10.3390/jfb14020090 (PMC9963507; doi:10.3390/jfb14020090)
Supplement: Supplementary file 1 [file jfb-14-00090-s001.zip › jfb-2135764-supplementary.pdf]

# *Supplementary Materials*: Fluorescent Carbon Dots from Food Industry By-Products for Cell Imaging

**Federica Mancini [1], Arianna Menichetti [2], Lorenzo Degli Esposti [1], Monica Montesi [1], Silvia Panseri [1], Giada Bassi [1,3], Marco Montalti [2], Laura Lazzarini [4], Alessio Adamiano [1] and Michele Iafisco [1,*]**

[1] Institute of Science, Technology and Sustainability for Ceramics (ISSMC), National Research Council (CNR), 48018 Faenza, Italy
[2] Department of Chemistry "Giacomo Ciamician", Alma Mater Studiorum-Università di Bologna, 40126 Bologna, Italy
[3] Department of Neuroscience, Imaging and Clinical Sciences, University of Studies "G. D'Annunzio", 66100 Chieti, Italy
[4] Institute of Materials for Electronics and Magnetism (IMEM), National Research Council (CNR), 43124 Parma, Italy
* Correspondence: michele.iafisco@istec.cnr.it

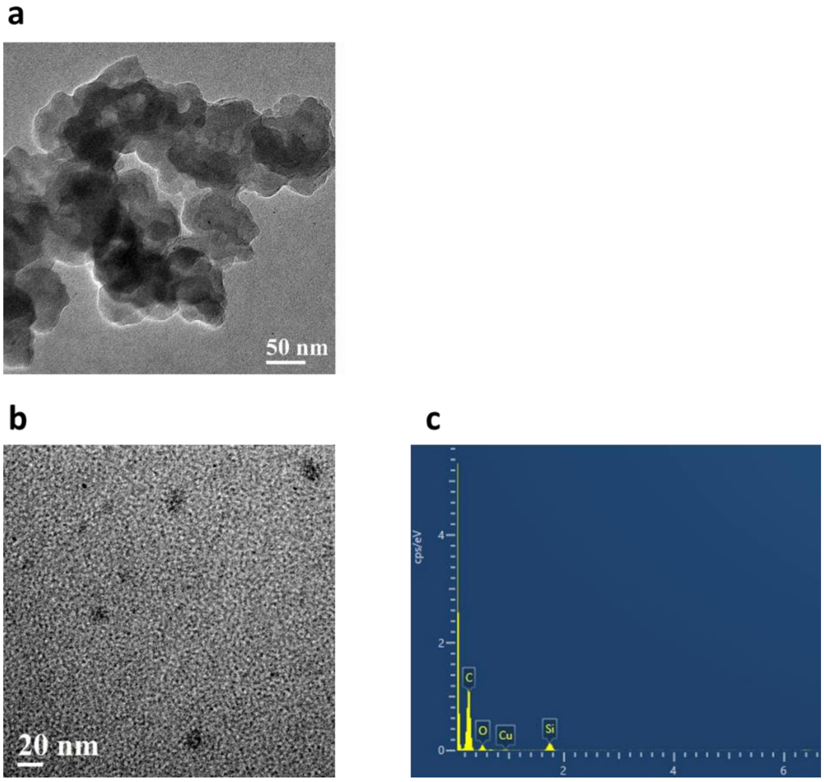


**Figure S1.** TEM micrographs of aggregated (a) and isolated (b) C-dots; EDS map of isolated carbon nanoparticles (c). There are no signals of impurities except for the Cu and Si ones, coming from the TEM chamber.

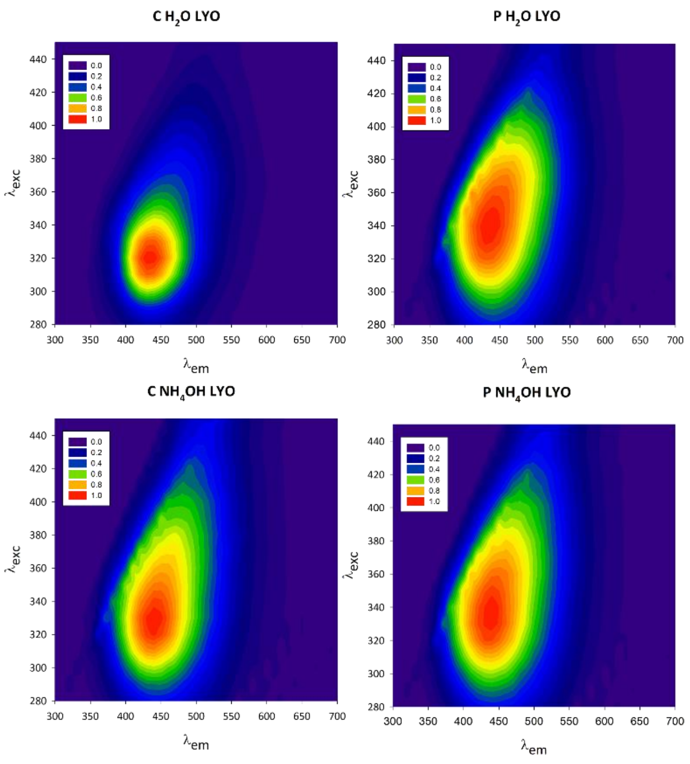


**Figure S2.** 3D excitation-emission maps of lyophilized C-dots.

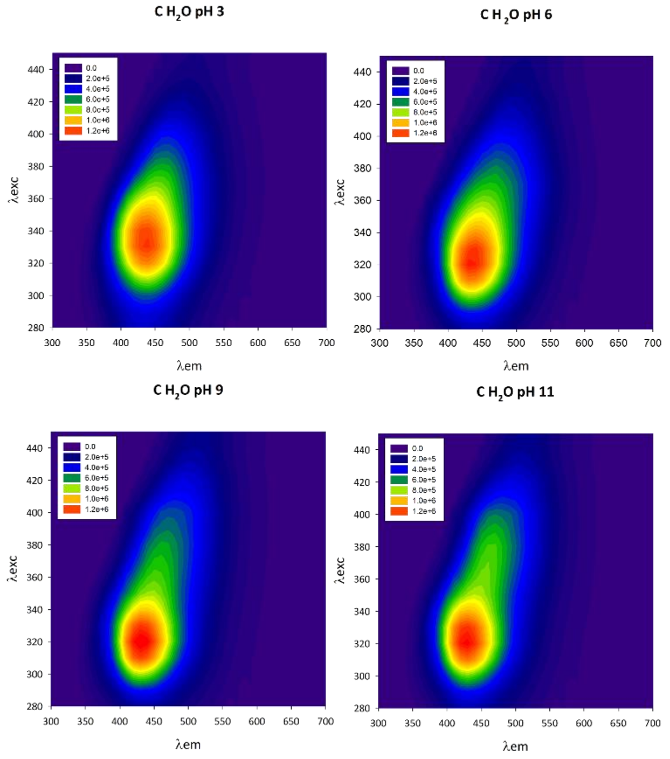


**Figure S3.** pH-dependent 3D excitation-emission maps of C $H_2O$.

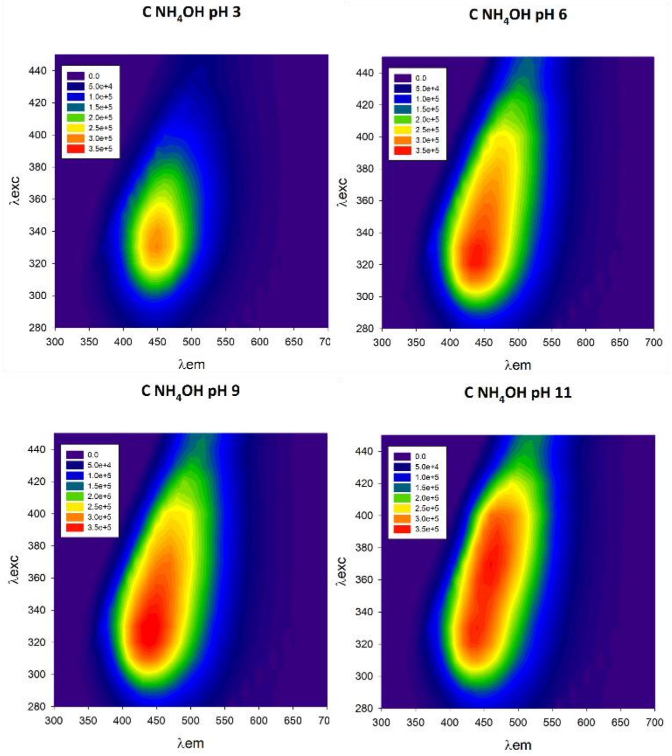


**Figure S4.** pH-dependent 3D excitation-emission maps of C $NH_4OH$.

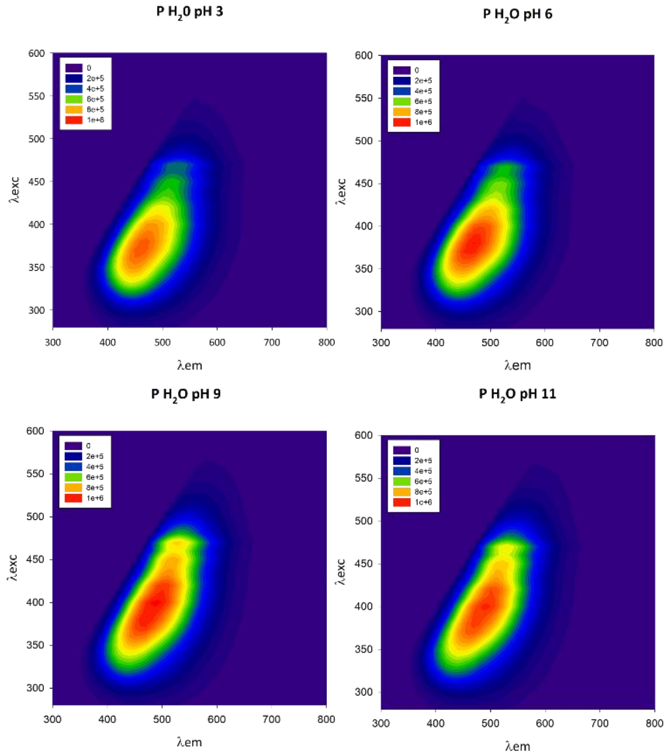


**Figure S5.** pH-dependent 3D excitation-emission maps of P $H_2O$.

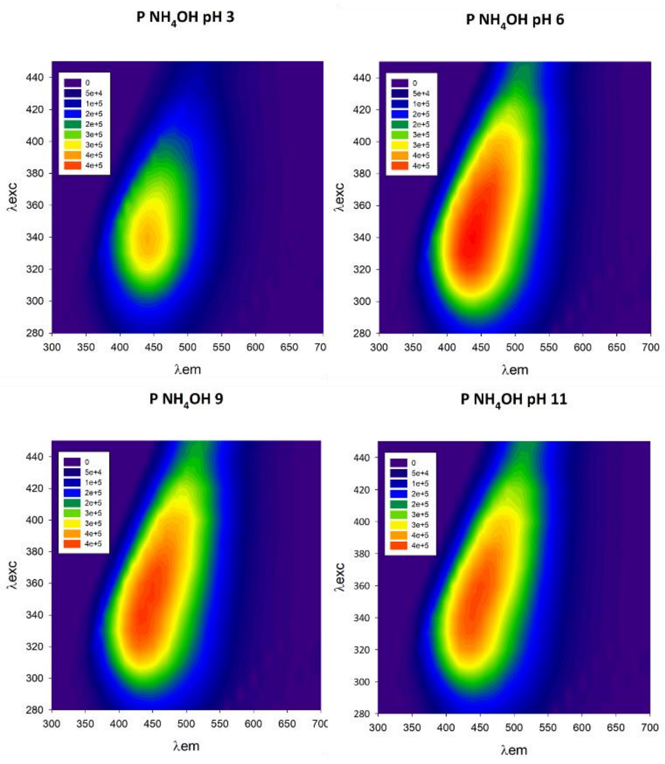


**Figure S6.** pH-dependent 3D excitation-emission maps of P $NH_4OH$.
